# Congenital Malformations of Calves Infected with Shamonda Virus, Southern Japan

**DOI:** 10.3201/eid2306.161946

**Published:** 2017-06

**Authors:** Yoshimasa Hirashima, Shoei Kitahara, Tomoko Kato, Hiroaki Shirafuji, Shogo Tanaka, Tohru Yanase

**Affiliations:** Kagoshima Central Livestock Hygiene Service Center, Hioki, Japan (Y. Hirashima, S. Kitahara);; National Agriculture and Food Research Organization, Kagoshima, Japan (T. Kato, H. Shirafuji, S. Tanaka, T. Yanase)

**Keywords:** Shamonda viruses, viruses, arbovirus, orthobunyavirus, Culicoides, infectious diseases, congenital malformations, central nervous system, meningitis/encephalitis, teratogenesis, arthrogryposis, calves, ruminants, Japan

## Abstract

In 2015 and 2016, we observed 15 malformed calves that were exposed to intrauterine infection with Shamonda virus, a Simbu serogroup orthobunyavirus, in Japan. Characteristic manifestations were arthrogryposis and gross lesions in the central nervous system. Our results indicate that this arbovirus should be considered a teratogenic virus in ruminants.

The Simbu virus serogroup is composed of >25 serologically related viruses in the family *Bunyaviridae*, genus *Orthobunyavirus* (*1*), which are transmitted mainly by *Culicoides* biting midges. Several of these viruses, such as Akabane virus, Aino virus, and Schmallenberg virus, are arboviruses associated with abortion, premature birth, stillbirth, and congenital malformations in ruminants (*2**–**4*).

The emergence and spread of Schmallenberg virus has had large socioeconomic effects in countries in Europe (*4**,**5*). Frequent epizootics of Akabane virus and Aino virus in Japan have caused many cases of congenital malformations in calves (*6*). However, the etiologic diagnosis for malformed calves associated with other arboviruses has not been established because of a lack of knowledge and sensitive diagnostic systems. Attempts to isolate viruses from sentinel cattle and *Culicoides* biting midges have contributed to knowledge about arboviruses circulating in nature and have, in some instances, helped predict the etiologic agents responsible for malformations (*7*).

Three Simbu serogroup viruses, Peaton virus, Sathuperi virus, and Shamonda virus (SHAV), were identified in Japan during the past 2 decades and have been suspected of being involved in congenital defects in calves (*8*). During December 2015–April 2016 in southern Japan, SHAV infections were identified in 15 malformed calves that had no antibodies against other teratogenic viruses. Of the 3 segments of the RNA genome of SHAV, the small and large segments have high genetic similarity with those of Schmallenberg virus, which implies the teratogenicity of SHAV in the ruminant fetus (*8*). Because there is no detailed description of an association between SHAV and malformations, we report details of these 15 clinical cases of malformations in calves suspected to be caused by SHAV infection.

## The Study

To obtain data on arboviruses circulating in 2015, we attempted to isolate viruses on BHK-21 and HmLu-1 cells inoculated with blood samples obtained from 60 sentinel cattle maintained on 30 farms and from pools of *Culicoides* biting midges collected by using suction light traps on 2 cattle farms in Kagoshima Prefecture in southern Japan. Two viruses (KS-1/P/15 and KS-2/P/15) were isolated from cattle blood collected during August and September 2015, and another virus (KSB-1/C/15) was isolated from a pool of *C. tainanus* midges sampled during September 2015.

We performed reverse transcription PCR (RT-PCR) with primer pairs (AKAI206F; 5′-CACAACCAAgTgTCgATCTTA-3′; and SimbuS637–656; 5′-gAgAATCCAgATTTAgCCCA-3′) specific for small RNA segment of Simbu serogroup viruses and the One Step RT-PCR Kit (QIAGEN, Hilden, Germany). We generated a product of the expected size from RNA samples of the isolated viruses. Preliminary sequence analysis for the RT-PCR product (443-nt) showed that the viruses were highly similar to SHAV. We sequenced and analyzed complete small and medium RNA segments and a partial region of the large RNA segment by using primers specific for SHAV (*8*). Sequences determined in this study were deposited in the International Nucleotide Sequence Database under accession nos. LC198185–93.

**Figure 1:**
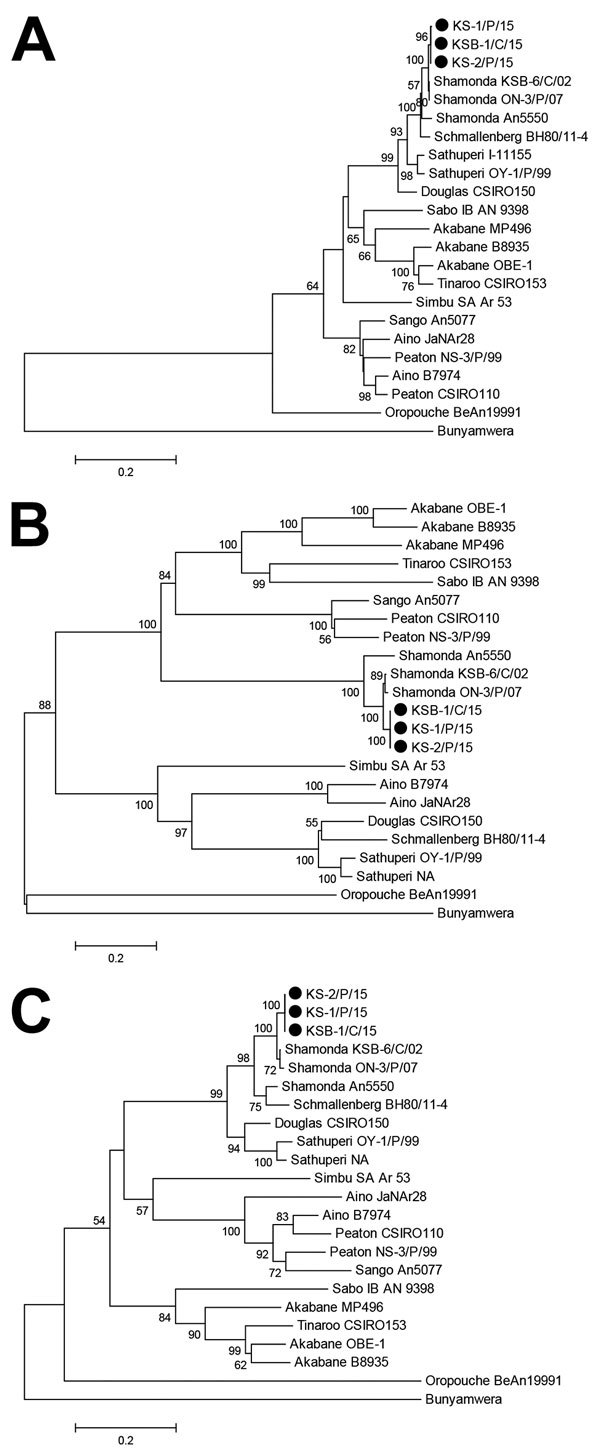
Neighbor-joining phylogenetic trees based on protein-coding sequences of A) small, B) medium, and C) large partial RNA segments for Simbu serogroup viruses, southern Japan, 2015–2016. Black circles indicate Shamonda viruses isolated in this study. Values along branches are percentages (≥50%) of bootstrap support of 1,000 pseudoreplicates. The 3 segmented RNAs of Bunyamwera virus were used as outgroups to root the trees. Scale bars indicate nucleotide substitutions per site. NA, details not available.

Neighbor-joining analysis available in MEGA7 (*9*) was used for phylogenetic analysis on the basis of the 3 RNA segments of the Simbu serogroup viruses. Sequences determined showed high nucleotide identities with known sequences of SHAV (98.3%–99.5% for the RNA small segment, 89.0%–97.9% for the medium RNA segment, and 91.5%–98.0% for the large RNA segment). Three phylogenetic trees showed that isolated viruses densely clustered with Japanese SHAV isolates obtained in 2002 and 2007 (Figure 1).

We performed virus neutralization tests (VNTs) on virus-infected HmLu-1 cells by using an established method (*2*). Antibodies to SHAV (titer range 1:2–1:64) were detected in serum samples from 15 malformed calves by VNTs during December 2015–April 2016 (Table). Serum samples obtained from sentinel cattle in June, August, September, October, and November 2015 showed that seroconversion for SHAV had occurred widely in Kagoshima Prefecture during August–October.

**Table:** Characteristics of 15 malformed calves infected with Shamonda virus, southern Japan, December 2015-April 2016*

Characteristic	Calf no.															Total
	1	2	3	4	5	6	7	8	9	10	11	12	13	14	15	
Gestational age, d	281	275	280	278	285	291	281	280	293	279	290	287	287	276	299	NA
Euthanasia	+	+	+	–	+	+	+	+	+	–	–	–	+	–	–	9
Stillbirth	–	–	–	+	–	–	–	–	–	+	+	+	–	+	+	6
RT-PCR result	+	–	–	+	+	+	–	–	–	+	–	+	–	–	+	7
Antibody titer	1:8	1:32	1:32	1:64	1:2	1:32	1:32	1:16	1:8	1:16	1:4	1:16	1:16	1:4	1:64	NA
Clinical finding																
Torticollis	+	–	+	+	+	–	+	+	–	+	–	+	–	+	+	10
Arthrogryposis	–	–	–	+	+	+	+	+	+	+	+	+	+	+	+	12
Macroscopic finding																
Head deformity																
Brachygnathism	+	+	+	–	–	–	–	–	–	–	–	–	+	–	–	4
Asymmetry of skull	+	–	+	–	–	–	–	–	–	–	+	+	–	–	–	4
LVE	–	–	–	–	–	–	–	–	–	+	–	+	–	–	–	2
Cerebellar hypoplasia	–	–	–	–	–	–	+	–	–	–	–	–	–	–	–	1
Spinal curvature	+	–	+	+	+	–	+	+	–	+	+	+	–	+	+	11
Muscle discoloration	–	–	–	–	+	+	–	–	+	+	+	–	–	–	–	5
Histopathologic finding																
Cerebrum																
Calcification of nerve cells	–	+	+	–	+	–	+	–	–	–	–	+	–	–	–	5
Brainstem																
Calcification of nerve cells	–	–	+	+	–	+	–	+	–	+	–	+	–	+	+	8
Perivascular infiltration	–	+	–	–	–	+	+	+	+	+	–	–	–	–	–	6
Gliosis	–	–	+	+	–	+	–	–	–	+	–	–	–	+	–	5
Spinal cord																
Decrease/disappearance of ventral horn cells	–	–	–	+	–	+	+	–	+	+	+	+	+	+	+	10
Skeletal muscles																
Fatty replacement	+	–	+	–	+	+	+	–	+	+	+	+	+	+	+	12
Atrophy	+	–	+	–	+	+	+	–	+	–	+	–	–	+	+	9
Myositis	+	–	+	–	+	+	+	–	+	–	+	–	–	+	+	9

*LVE, lateral ventricular enlargement; NA, not applicable; RT-PCR, reverse transcription PCR; +, positive; –, negative.

Although 2 calves ingested colostrum substitute containing immunoglobulins, all calves tested were deprived of colostrum produced by their mothers. SHAV had not been detected in mainland Japan for ≈10 years until we identified new cases of infection in 2015. It is highly improbable that the colostrum substitute contained antibodies against SHAV. Malformed calves were delivered at or beyond term (gestation periods range 275–299 days), but 6 calves were born dead.

The small RNA–specific RT-PCR showed positive results for samples from the central nervous system (cerebrum, brainstem, or spinal cord) of 7 deformed calves. Sequences obtained were identical (except for 1 of 2 nt substitutions) with relevant sequence of SHAVs isolated in 2015.

**Figure 2:**
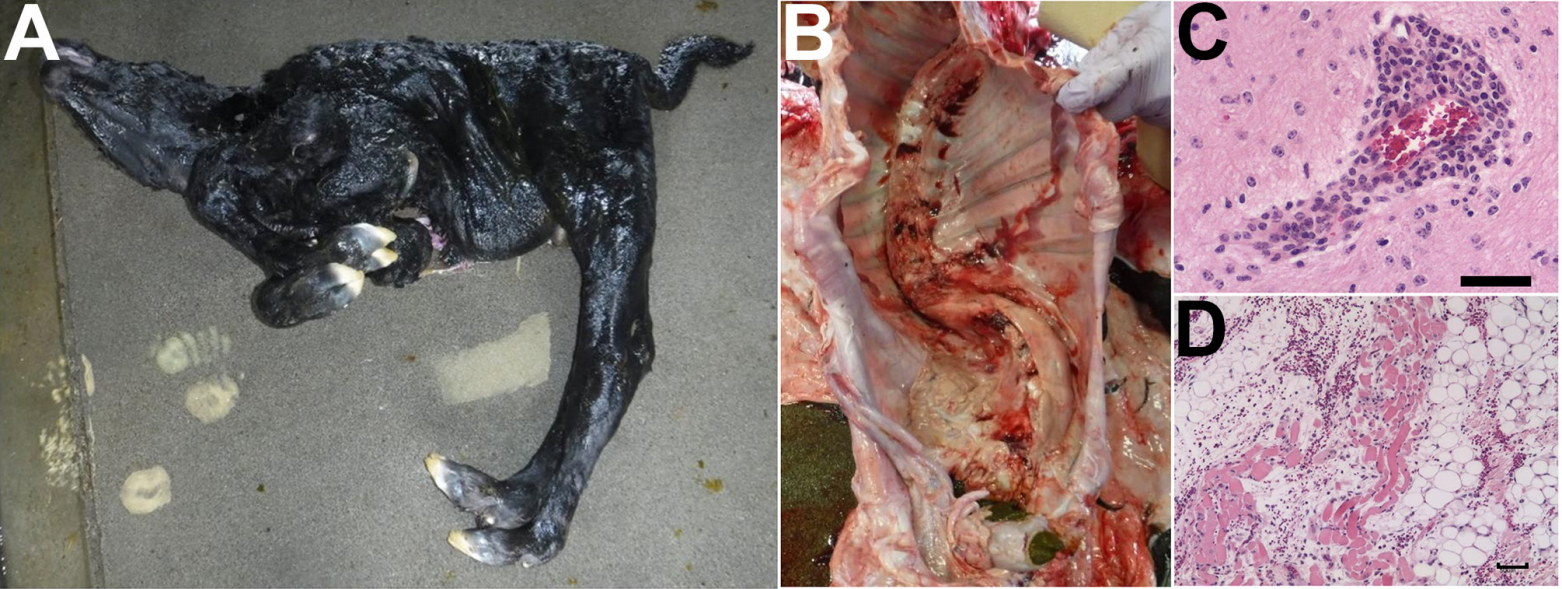
Characteristic observations in Shamonda virus–positive malformed calves, southern Japan, 2015–2016. A) Torticollis and arthrogryposis in calf 3. B) Spinal curvature (scoliosis) in calf 7. C) Perivascular infiltration in the midbrain of calf 7. D) Fatty replacement and atrophy in skeletal muscle of calf 3. For histopathologic analysis, thin sections prepared from paraffin-embedded tissues were stained with hematoxylin and eosin. Scale bars indicate 50 μm.

Torticollis (10/15) or arthrogryposis (12/15) were often observed among affected calves (Figure 2). Head deformity (6/15) or spinal curvature (11/15) were also characteristic. Discoloration of skeletal muscles was observed in one third of the calves. Cerebral hemispheres appeared normal among the calves, but lateral ventricular enlargement was found in 2 calves, and cerebellar hypoplasia was found in 1 calf.

Histopathologic analysis identified calcification of nerve cells (11/15), perivascular infiltration of mononuclear cells (6/15), and gliosis (5/15), which were often observed in the cerebrum and brainstem (Figure 2). Severe degenerative changes in the ventral horn of the spinal cord were identified in 10 of the malformed calves. Fatty replacement (12/15), atrophy (9/15), and myositis (9/15) were major observations in the skeletal muscle and often correlated with muscle discolorations.

VNTs did not detect neutralizing antibodies against teratogenic arboviruses, such as Chuzan virus, Akabane virus, Aino virus, or Peaton virus in serum samples from affected calves. All tested dams of affected newborns were positive for SHAV antibodies in VNTs (titer range 1:16–>1:256).

## Conclusions

Our findings support an association between SHAV and congenital deformities in calves infected in utero. Manifestations and macroscopic and microscopic observations in the malformed calves were similar to those of calves infected with Schmallenberg virus (*10**,**11*). In comparison with cases attributed to Akabane virus and Aino virus, congenital lesions in the brain were relatively mild (i.e., no hydranencephaly and few lateral ventricular enlargements appeared in malformed calves). However, nonsuppurative encephalitis or nerve cell death often occurred in cerebrums and brainstems that otherwise seemed normal.

Twelve of the dams of the malformed calves in this report were vaccinated with a trivalent inactivated vaccine containing Akabane, Aino, and Chuzan viruses. To our knowledge, no effective preventive measure for infection with SHAV is available. Previous surveillance in Africa, the Middle East, and Asia (*12**–**14*) enabled us to postulate the wide geographic distribution of SHAV. The potential risk for SHAV spreading in livestock should be considered, even in previously unaffected areas, because long-distance dispersal and accidental transportation of infected vectors from epizootic areas can introduce the virus. Also, recent outbreaks of infection with Schmallenberg virus and SHAV suggest that many Simbu serogroup viruses can affect livestock. More detailed study of this virus serogroup is warranted.
